# In situ splitting after selective partial portal vein ligation or simultaneous hepatic artery ligation promotes liver regeneration

**DOI:** 10.1038/s41598-018-26742-5

**Published:** 2018-06-07

**Authors:** Li-Bin Yao, Chong-Hui Li, Xiao-Juan Wu, Xue-Dong Wang, Xin-Lan Ge, Ai-Qun Zhang, Xiao-Cheng Zhu, Yong Shao, Jia-Hong Dong

**Affiliations:** 1 The Center for Hepatopancreatobiliary Diseases, Beijing Tsinghua Changguang Hospital, Tsinghua University Medical Center, Litang Road 168, Beijing, 102218 P.R. China; 2 Department of General Surgery, the Affiliated Hospital of Xuzhou Medical University, Huaihaixi Road 99, Quanshan District, Xuzhou, 221000 Jiangsu P.R. China; 3 Institute of Hepatobiliary Surgery, Chinese PLA General Hospital, Chinese PLA Medical School, Fuxing Road 28, Beijing, 100853 P.R. China; 4 Department of Nephrology, Huai’an Hospital Affiliated to Xuzhou Medical University and Huai’an Second Hospital. Qingpu District, Huai’an, 223000 Jiangsu P.R. China

## Abstract

This study seeks to compare the impact of selective partial portal vein ligation (PPVL) or the combination of simultaneous hepatic artery ligation (PPVAL) with *in situ* splitting (ISS) on liver regeneration and injury. Rats were randomized into three groups; namely: selective PVL, PPVL + ISS and PPVAL + ISS. The changes in hepatic hemodynamics, liver regeneration and hepatocytic injury were examined. Blood flow to the left portal branch and the microcirculation of the left median lobe after PPVL or PPVAL was significantly reduced. Liver regeneration of PPVAL + ISS group was more pronounced than that in the PPVL + ISS and PVL groups at 48 and 72 hours as well as 7 d postoperatively. The serum biochemical markers and histopathological examination demonstrated reduced levels of liver injury in the PPVL + ISS group. Injury to hepatocytes was more pronounced with PPVAL + ISS than PVL. HGF, TNF-α and IL-6 expression in the regenerated lobes in both PPVAL + ISS and PPVL + ISS groups increased significantly when compared to the PVL group. We demonstrated that both PPVL + ISS and PPVAL + ISS were effective and feasible means of inducing remnant liver hypertrophy and could serve as a rapid clinical application for qualified patients.

## Introduction

Hepatectomy is considered one of the most effective treatments for liver tumors of primary or secondary origin. However, in advanced tumors, R0 resection usually results in inadequate future liver volume causing liver failure which restricts its application^1,2^. Portal vein embolization (PVE) was originally introduced to clinical practice by Makuuchi *et al*. in 1980s^3^; this procedure induced hypertrophy of the non-occluded liver segments and atrophy of the occluded liver segments by embolizing the right or left main branch of the portal vein as appropriate. This procedure not only allowed patients who required major liver resection but had an insufficient estimated functional residual liver volume to avoid postoperative liver failure and to have access to the opportunity of surgery as well as obviously improved the long-term survival of these patients.

However, some shortcomings and disadvantages of this procedure have been noted in the increasing application of preoperative PVE or portal vein ligation (PVL). Focal hepatic necrosis or even liver abscess may occur in occluded lobes when portal branches are embolized or ligated. Recent studies have shown that the future remnant liver volume could increase from 8% to 46% between 2 and 8 weeks after PVE/PVL^4–6^. That is, the sufficient remnant liver volume is not always achieved using PVE or PVL.

In addition, the mean period between PVE and liver surgery was 36.9 days according to the literature^7^. These studies provided the temporal conditions required for tumor growth, invasion and metastasis. Moreover, numerous clinical and animal experimental reports also showed that PVE/PVL enhanced tumor progression in the occluded lobe, which may result in abandoning the original plan of extended hepatectomy because the future remnant liver or main hepatic vessels were invaded due to rapid tumor growth or extrahepatic metastasis occurred^5,8^. One of the main reasons of rapid tumor growth after PVE or PVL was alteration in hepatic blood supply according to the literature. Compensatory arterial perfusion increased after reduction of segmental portal blood flow, which is known as the hepatic arterial buffer response^9–14^. Clinical and experimental studies have demonstrated a significant increase in hepatic arterial blood flow in the occluded liver lobes resulting from an increase in common hepatic arterial flow. Increased hepatic arterial blood flow provides a richer blood supply for tumor in the occluded lobe and thus promote rapid tumor growth, especially for primary hepatocellular carcinoma, which mainly depends on the arterial blood flow as the main blood supply^15^.

ALPPS attracted worldwide attention and discussion in the field of hepatobiliary surgery because of its amazing extent of liver regeneration and obviously shortened waiting time before the second surgery^16,17^. However, ALPPS can cause even more serious necrosis of occluded lobes and potentially promote tumor growth in the occluded lobe^18^. Bilodeau and his colleagues confirmed that selective partial portal vein ligation could induce contralateral lobe hypertrophy and reduce the injury or necrosis of the ipsilateral lobe by studying of different degrees of selective partial portal vein ligation in rats^19^. Necrosis was found only when the degree of ligation was severe.

Because *in situ* splitting could promote obvious remnant liver regeneration, we assume that this procedure may induce rapid remnant liver regeneration, ameliorate liver injury and inhibit tumor progression in the occluded lobe when combined with either partial portal vein ligation or simultaneous hepatic artery ligation (HAL) of the occluded liver lobe. To confirm this hypothesis, a rat model of partial PVL with or without simultaneous HAL and combined with ISS was established in this study. Then, we compared the hepatic regeneration rate of the remnant liver lobe and the degree of occluded lobe necrosis in the different treatment groups and evaluated the potential clinical value of *in situ* splitting in order to develop a theory and new ideas for solving problems related to postsurgical complications of PVE/PVL.

## Materials and Methods

### Animals

Male Sprague-Dawley rats with body weight of 250–280 g were obtained from the Animal Center of the Academy of Military Medical Science of PLA in China. Animals were housed for 12 h light and dark cycle without disease-specific pathogens conditions and allowed access to standard rodent chow and water for ad libitum. The procedures of animal experiments were approved by the ethics committee of animal study in Chinese PLA General Hospital. All experiments were performed in accordance with the recommendations in the Guide for the Care and Use of Laboratory Animal of the National Institutes of Health.

### Experimental groups and surgical procedures

The animals were randomly divided into the following 3 experimental groups according to different procedures: selective portal vein ligation (PVL), selective partial portal vein ligation combined with *in situ* splitting (PPVL + ISS) and selective partial portal vein ligation with simultaneous hepatic artery ligation combined with *in situ* splitting (PPVAL + ISS). All rats were fasted overnight before surgery and anesthetized using inhalation anesthesia. A binocular operating microscope was used for all operations. The abdominal incision was closed by double layer running suture after surgery. The following paragraph describes the specific surgical procedures for each group in detail.

**Figure 1:**
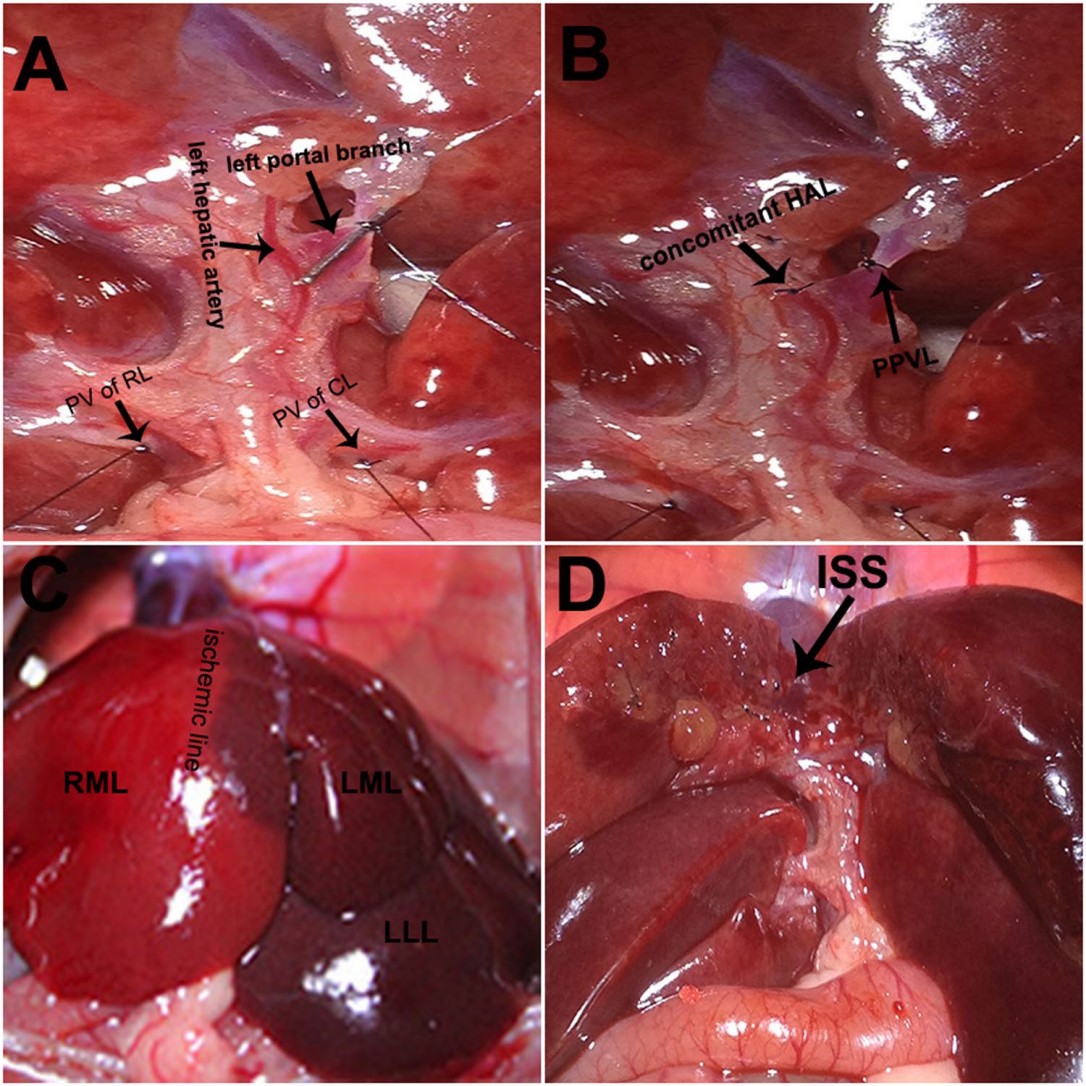
Surgical procedures of PPVAL + ISS. (**A**) The portal branches of the caudal lobe (CL) and the right lobe (RL) were ligated, and the left portal branch was ligated with a needle. (**B**) The needle was removed, and the concomitant hepatic artery of the left lateral and left median lobes (i.e., the left hepatic artery) was ligated. (**C**) An ischemic line emerged at the right of the falciform ligament after PPVAL. (**D**) *In situ* splitting (ISS) was performed along the border between the left median lobe (LML) and the right median lobe (RML). (Left lateral lobe, LLL. Portal vein, PV).

Group 1, PVL: The corresponding portal veins of the right lobe, caudal lobe, left median and left lateral lobes were ligated, and the right median lobe was preserved for regeneration. Group 2, PPVL + ISS: The corresponding portal veins of the right lobe and the caudal lobe were ligated, the left portal branch of the median lobe was partially ligated, and ISS was carried out along the ischemic line between the right median lobe and the left median lobe. Group 3, PPVAL + ISS: The hepatic artery feeding the left median and left lateral lobes was ligated and cut off after selective PPVL as described in Group 2, and then *in situ* splitting was performed. The blood flow through the portal trunk of left lateral and left median lobes was restricted through PPVL according to the method described by Vorobioff *et al*.^20^. Briefly, the left portal branch was carefully separated from the concomitant hepatic artery. The isolated portal branch was ligated with a needle (diameter: 0.4 mm) and 6-0 silk, and then the needle was removed. The choice of the needle diameter was based on our preliminary experiments on the degree of regeneration of non-occluded lobes and necrosis of occluded lobes in response to different needle diameters of needle while performing PPVL with or without simultaneous hepatic artery ligation combined with *in situ* splitting. The mean diameter of the left portal branch in 15 rats weighting 250–280 g was 1.28 ± 0.05 mm. The ISS was performed according to the method described in our previous study^18^. The general surgical procedure is shown in Fig. 1.

Six rats in each group were sacrificed at each time point (24 h, 48 h, 72 h and 7 d). The blood samples were drawn from the inferior vena cava at different time points and immediately centrifuged at 3,000 rpm for 10 min, then the serum were stored at −80 °C until analyses for biochemical detection. The total liver lobes were removed and divided into the right lobe, caudal lobe, left lateral lobe, left median lobe and right median lobe. The lobes were weighed using a laboratory electronic microscale, and then approximately 200 mg of liver tissue from the left and right median lobes was immediately put into liquid nitrogen and then moved to −80 °C refrigerator. The remaining liver tissues were fixed in 10% formaldehyde.

### Detection of portal blood flow with laser speckle contrast imaging

An ultrasound flowmeter (Transonic Systems Inc. TS420. USA) and laser speckle contrast imaging (LSCI, Moor instruments, UK) were used to detect the blood flow through the left portal branch and the microcirculatory blood perfusion, respectively, of the left median and right median lobes either before and after PPVL or HAL during surgery. A total of 8 rats randomly selected during surgery were used to detect the blood flow through the left portal branch at 5 min after laparotomy, PPVL and HAL as appropriate. The abdominal cavity was filled with warm saline, and the left portal branch was maintained tension-free in the probe during the measuring process of the ultrasound flowmeter. We also randomly selected six rats during surgery to compare the microcirculatory blood perfusion of the left median and right median lobes before and after PPVL and HAL as appropriate. The specific measurement of LSCI was the same as the method introduced in our previous study^18^.

### Hepatic regeneration rate

The hepatic regeneration rate (HRR) of the right median lobe was calculated according to our previous study^18^. Briefly, HRR = (W_A_ − W_I_)/W_I_ × 100%. W_A_: The weight of hyperplastic right median lobe, W_I_: The weight of initial right median lobe before surgery.

### Detection of serum biochemical markers

The serum biochemical markers including aspartate aminotransferase (AST), alanine aminotransferase (ALT), total bilirubin (TBIL) and albumin (ALB) were analyzed with an automatic biochemical analyzer (Cobas-Mira Plus, Roche, Manheim, Germany) in the Clinical Biochemistry Department.

### Histological analysis

Liver tissue specimens were stained with hematoxylin and eosin (HE) after immersion fixed in 10% formaldehyde. Immuno-staining of all liver sections of the right median lobe was done for Ki-67 identification as per instruction manual (mouse monoclonal antibody Ki-67, BD Biosciences, USA). After counter-staining of all immune-stained tissue sections with hematoxilin. Identification of Ki-67 positive hepatocytes was done by random selection of 5 visual fields (200×) and 10 random visual fields (100×) were used to quantify necrotic areas in left medial lobes using Adobe Photoshop CS5. Necrosis was scored based on Veteläinen R *et al*.’s^21^ scoring system. 0, no necrosis; 1, less than 25%; 2, 25–50%; 3, 50–75%; and 4, at least 75% necrosis. All histological analysis was done by single pathologist who was blind to experimental groups.

### RT-PCR of TNF-α, IL-6, HGF and HSP70

TRIzol reagent (Invitrogen Life Technologies, Carlsbad, CA, USA) was used for extracting total RNA from liver tissue for spectro-photometrical quantification. RevertAid First Strand cDNA Synthesis Kit and oligo-dT primers (Thermo Fisher Scientific Ins, Burlington, ON, Canada) were used for reverse transcription of RNA into cDNA. For the expression of tumor necrosis factor-α (TNF-α), interleukin-6 (IL-6), hepatocyte growth factor (HGF) and heat shock protein 70 (HSP70)^18^, specific primers were designed and rat glyceraldehyde-3-phosphate dehydrogenase (GAPDH) was used as endogenous control. RT-PCR detection system (Applied Biosystems AB, USA) was used for quantitative real-time PCR amplification with a SYBR PCR Kit (TaKaRa Bio, Inc, Dalian, China). PCR amplification was performed with the following conditions: 1 cycle of 95 °C for 30 s; 40 cycles of 95 °C for 5 s, 55 °C (for GAPDH, HGF and HSP70) or 58 °C (for TNF-α and IL-6) for 15 s, and 72 °C for 15 s. 2^−ΔΔCt^ method^22^ was used for calculating relative mRNA expression. The results represent an x-fold induction versus the baseline levels in the PVL group.

### Statistical analysis

All experimental data are expressed as the means ± SD. The data of the groups were compared using *t*-test, Mann-Whitney U test and one-way analysis of variance (ANOVA), *p* < 0.05 was considered as a significant difference. Statistics were calculated using SPSS software (SPSS Inc, version 18.0, Chicago, IL).

### Data availability statement

The datasets generated during and/or analysed during the current study are available from the corresponding author on reasonable request.

## Results

### Preliminary experiments showed a needle 0.4 mm in diameter was suitable for performing PPVL in rats

In our preliminary experiment, we first used 0.6 mm and 0.5 mm diameter needles to restrict the blood flow through the left portal branch. We observed that the right median lobes in the PPVAL + ISS group showed a very low HRR (data not shown) at 72 h after surgery, and the left lobe only had occasional mild necrosis in random spots. When we used 0.4 mm and 0.25 mm diameter needles to further restrict the blood flow of the left portal branch, the right median lobes HRR was significantly increased. However, severe hepatic necrosis and hepatic abscess occurred in the left lobe (PPVAL lobe) when PPVAL + ISS was carried out using a 0.25-mm diameter needle, while there was only spotty necrosis or occasional small patchy necrosis on the surface of the left hepatic lobes and no severe hepatic necrosis or abscess when PPVAL + ISS was performed using a 0.4-mm diameter needle. Therefore, we selected the 0.4-mm diameter needle for PPVL + ISS or PPVAL + ISS in the subsequent experiments.

### Microcirculation of the median lobes and blood flow through the left portal branch before and after PPVL and HAL blood flow controls

**Figure 2:**
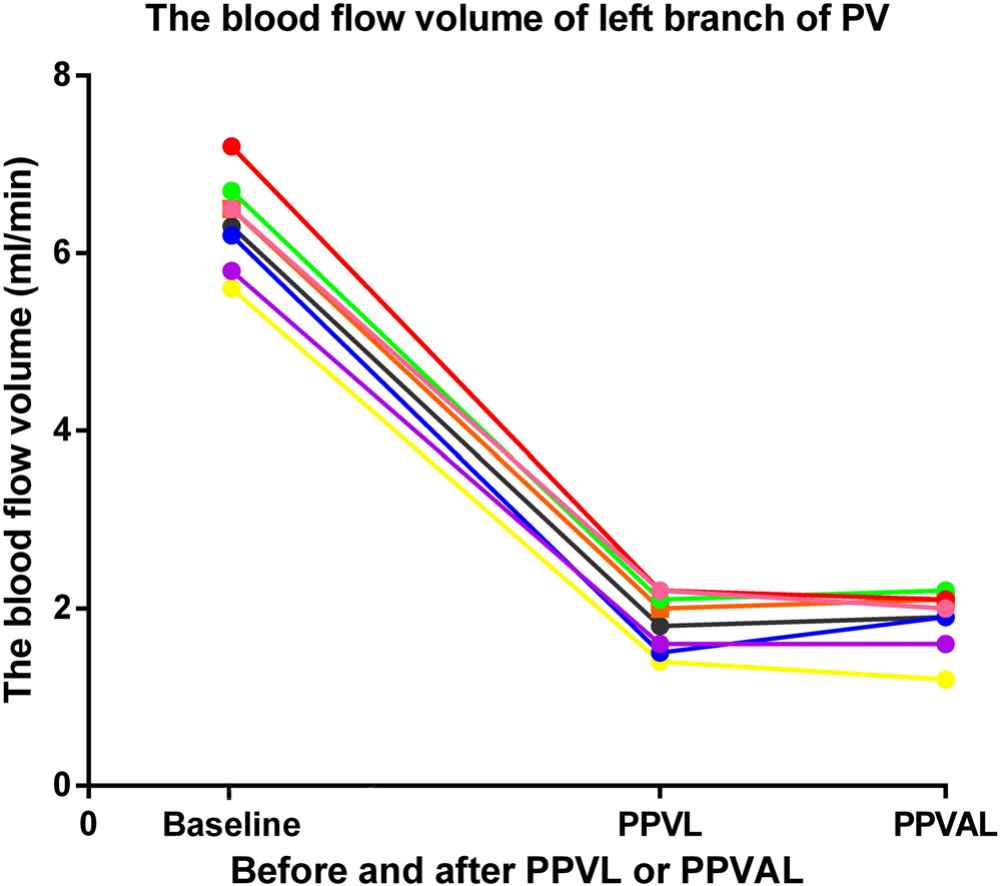
The blood flow through the left portal branch before and after PPVL or PPVAL. The blood flow through the left portal branch was obviously decreased after PPVL and showed no obvious change before and after PPVAL.

We detected the blood flow changes through the left portal branch from baseline to after PPVL and HAL in the control animals. The results showed that the blood flow through the left portal branch was 6.35 ± 0.50 ml/min before PPVL and was reduced to 1.85 ± 0.32 ml/min after PPVL, and the difference was significant (*p* < 0.01). The blood flow of left portal branch following PPVL decreased to 29% of the baseline value, and the degree of restriction of the left portal branch blood flow was approximately 71% when using a 0.4-mm diameter needle. The blood flow was 1.88 ± 0.33 ml/min after the concomitant hepatic artery ligation, and no significant difference was found with and without concomitant hepatic artery ligation **(**Fig. 2**)**.

**Figure 3:**
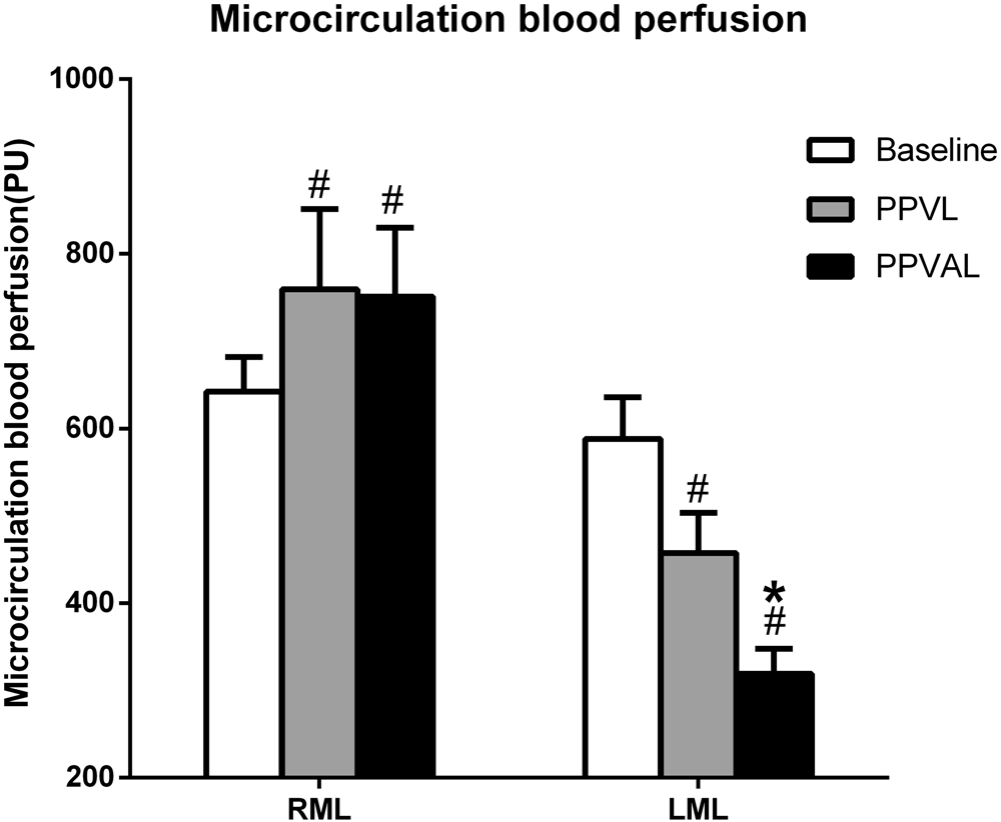
The microcirculatory blood perfusion of the median liver lobes. The microcirculatory blood perfusion of the right median lobes (RMLs) was higher after PPVL or PPVAL than at baseline, and no significant change was observed after HAL (^#^*p* < 0.05 compared with baseline). The microcirculatory blood perfusion of the left median lobes (LMLs) was lower after PPVL or PPVAL than at baseline, and that of the left median lobes was obviously decreased after HAL (*p < 0.05 compared with PPVL).

The results of the LSCI measurement showed that the microcirculatory blood perfusion of the right median lobes increased from 641.83 ± 39.66 PU to 759.27 ± 92.10 PU and that of left median lobes decreased from 588.37 ± 47.27 PU to 457.52 ± 45.95 PU after PPVL (*p* < 0.05). The microcirculatory blood perfusion of the left median lobes further decreased to 318.98 ± 28.50 PU after HAL, which was obviously lower than before HAL (*p* < 0.05). However, the microcirculation of the right median lobes did not change significantly after concomitant hepatic artery ligation **(**Fig. 3**)**.

### PPVAL + ISS accelerated the regeneration of the right median lobes

**Figure 4:**
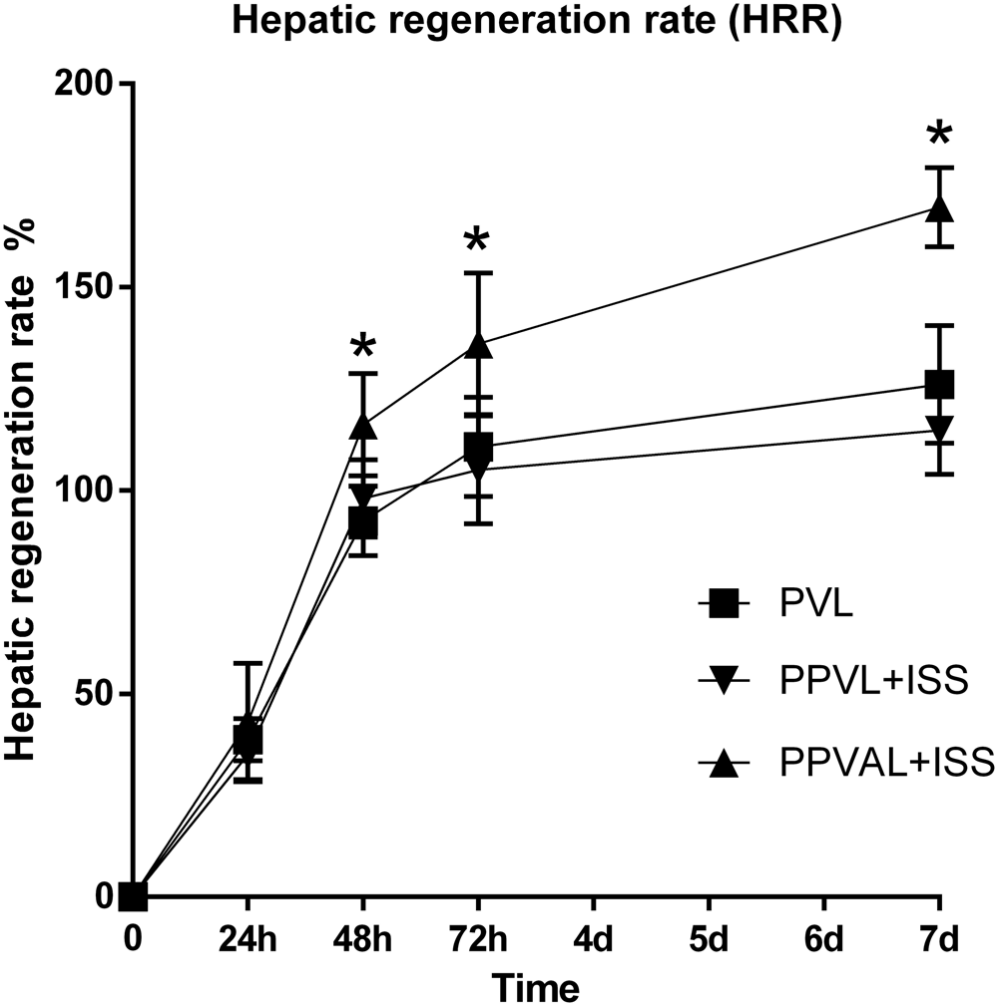
The hepatic regeneration rate (HRR) of the right median liver lobes of the different groups. The HRR of the PPVAL + ISS group was higher than that of the PPVL + ISS and PVL groups at 48 h, 72 h and 7 d (*p* < 0.05), and no significant differences were observed between the PPVL + ISS and PVL groups at all time points. *p < 0.05 compared with PPVL + ISS or PVL.

In order to observe the effects of PPVL + ISS and PPVAL + ISS on liver regeneration, we detected the HRR and Ki-67 expression in the right median lobes. The HRRs of the three groups showed no significant differences at 24 h after surgery. Comparing the HRR of the PPVAL + ISS group to the PPVL + ISS and PVL groups at 48 h, 72 h and 7 d after surgery, we found that HRR was significantly higher in the PPVAL + ISS group (*p* < 0.05). Additionally, although the HRR at 48 h and 7 d after surgery was lower in the PPVL + ISS group than in the PVL group, there were no significant differences between the PPVL + ISS and PVL groups at all time points **(**Fig. 4**)**.

**Figure 5:**
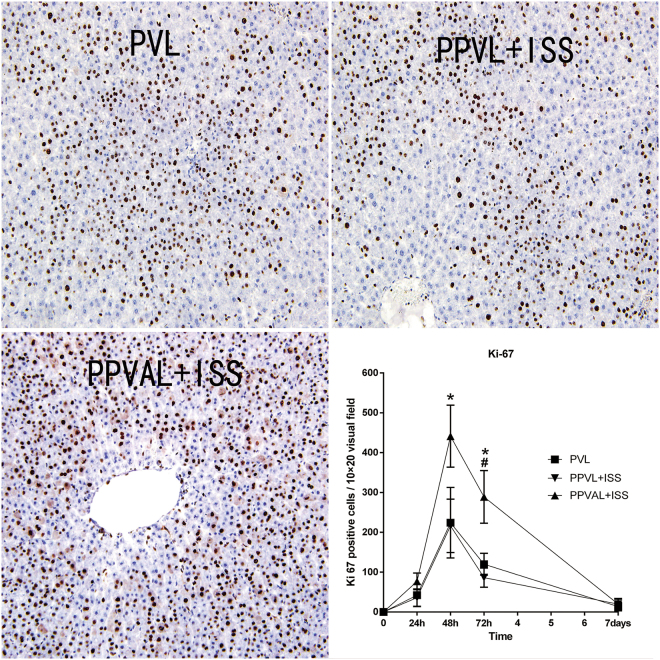
The number of Ki-67-positive hepatocytes in the regenerating liver lobes. (**A**) The expression of Ki-67 in regenerating lobes at 48 h after surgery in each group. (**B**) The number of Ki-67-positive hepatocytes in the regenerating liver lobe shows a significant difference at 48 h and 72 h. There was no significant difference at 24 h and 7 d. *p < 0.05 indicates PPVAL + ISS compared with PPVL + ISS and PVL. ^#^*p* < 0.05 indicates PPVL + ISS compared with PVL.

To verify the above results, we measured the Ki-67 expression (a nuclear antigen associated with hepatocyte proliferation) in the right median lobes. At 24 h and 7 d after surgery, Ki-67 expression in the regenerating liver lobes (i.e., the right median lobes) showed no obvious differences in all the groups. However, the number of hepatocytes positive for Ki-67 in the regenerating lobes was significantly greater in the PPVAL + ISS group compared to PVL and PPVL + ISS groups at 48 h (*p* < 0.05) and 72 h (*p* < 0.05). In addition, the number of positive hepatocytes for Ki-67 was greater in the PVL group than in the PPVL + ISS group at 72 h (*p* < 0.05) **(**Fig. 5**)**.

### PPVAL + ISS aggravated injury and necrosis of the occluded lobes

**Figure 6:**
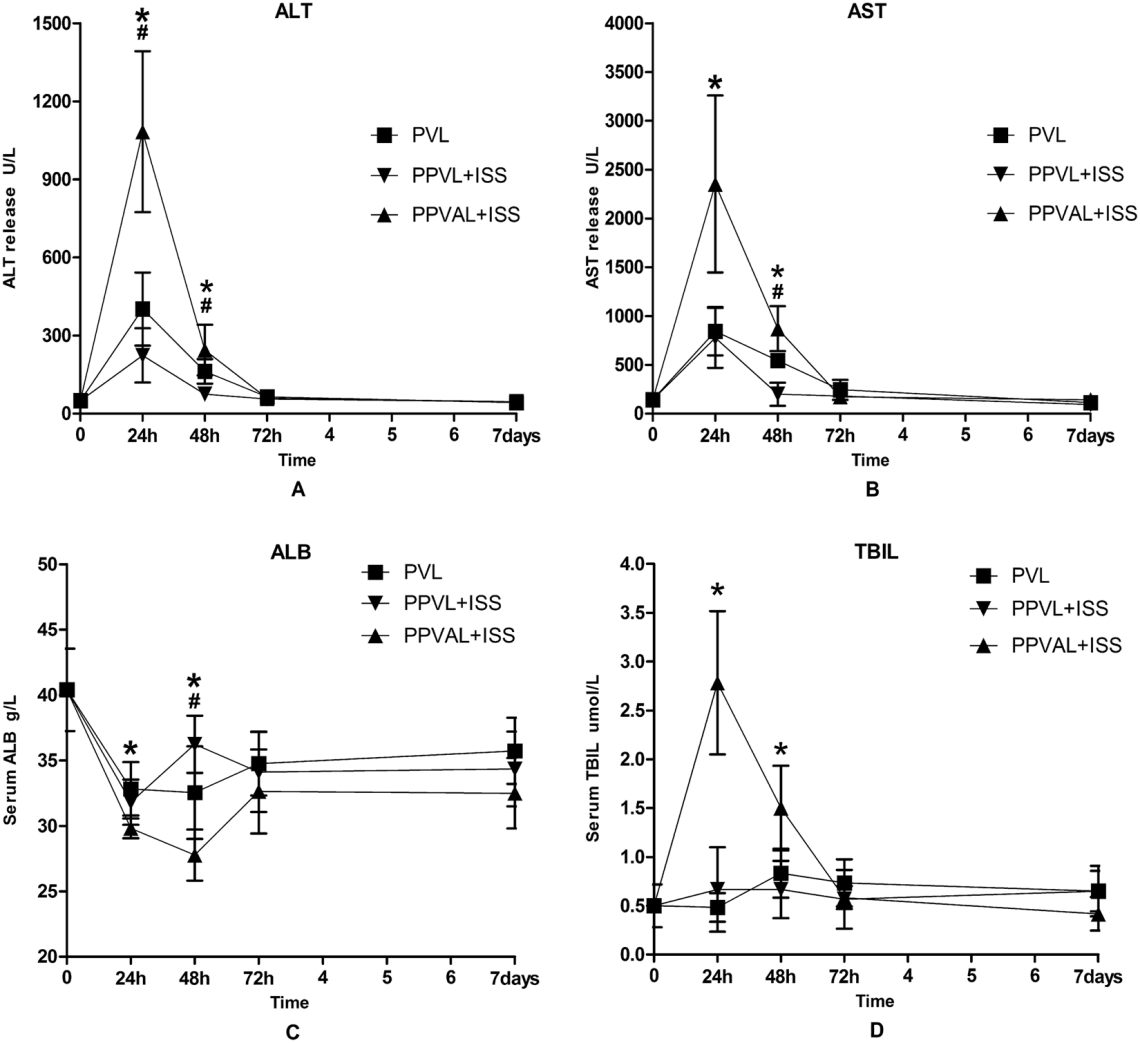
Serum biochemical markers after different surgical interventions. Values are presented as the means ± SD. There was a significant difference in the ALT and AST levels between the PPVAL + ISS group and either the PPVL + ISS or PVL groups at 24 h and 48 h after surgery (*p* < 0.05), but no significant difference was observed at 72 h and 7 days. The serum ALB levels of the PPVAL + ISS group were significantly different at 24 and 48 h compared with the levels of the PPVL + ISS and PVL groups (*p* < 0.05). The serum levels of TBIL in the PPVAL + ISS group were higher than those in the PPVL + ISS and PVL groups at 24 h and 48 h (*p* < 0.05). None of the serum biochemical markers showed a significant difference at 72 h and 7 d. *p < 0.05 compared with PPVL + ISS or PVL. ^#^*p* < 0.05 compared with PVL.

In order to determine the extent of the damage induced by different blood occluding procedures on the left lobe (i.e., the occluded lobe), we measured serum ALT, AST, ALB and TBIL levels and performed HE staining. Hepatic enzymes (ALT and AST) levels at 24 h after surgery in the PPVAL + ISS group were significantly higher compared to PPVL + ISS and PVL groups (*p* < 0.05). ALT levels in the PPVL + ISS group differed significantly when compared to the PVL group (*p* < 0.05), but AST levels did not differ at 24 h between these two groups. Levels of ALT and AST in the PPVAL + ISS group at 48 h after surgery were still significantly higher when comparing with PPVL + ISS group and PVL group (*p* < 0.05); meanwhile, levels of ALT and AST in the PPVL + ISS group were obviously lower than those in the PVL group (*p* < 0.05). No significant differences were observed among all groups at 72 h and 7 d after surgery. Serum ALB concentration of PPVAL + ISS group was lower than those in the PVL and PPVL + ISS groups at 24 h and 48 h (*p* < 0.05), and there was no statistically significant difference between the PVL group and PPVL + ISS group 24 h after surgery (*p* > 0.05). In the PVL group, serum ALB level at 48 h was lower compared to the PPVL + ISS group (*p* < 0.05). No significant differences among the three groups were observed at 72 h and 7 d after surgery. Serum TBIL levels at 24 h and 48 h after surgery were significantly higher in the PPVAL + ISS group comparing with PVL group and PPVL + ISS group (*p* < 0.05). No significant differences were observed among all groups when serum TBIL was measured at other time points (*p* > 0.05) **(**Fig. 6**)**.

**Figure 7:**
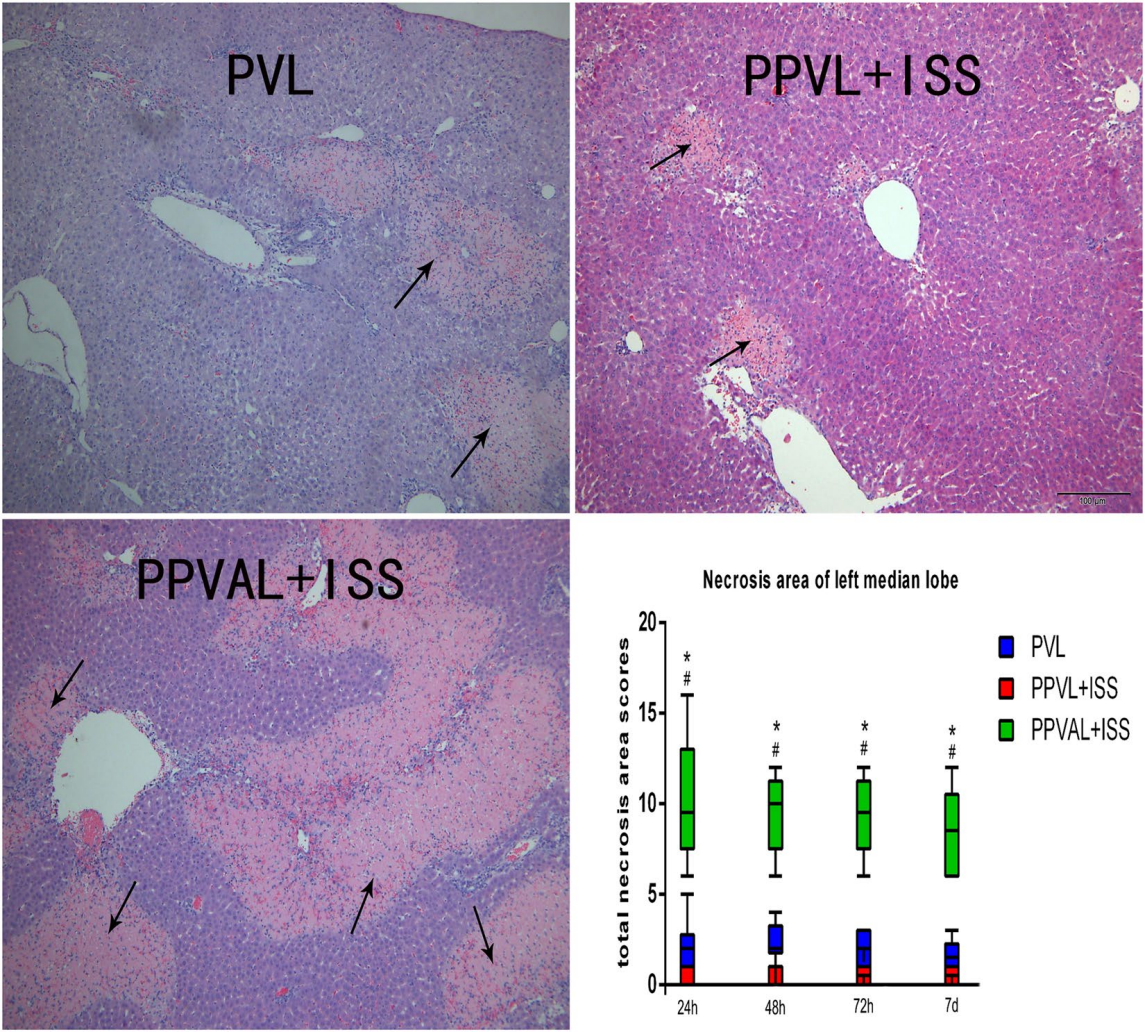
Hepatocytic necrosis in the left median liver lobes at all time points. HE staining shows necrosis of the left median lobes from each group at 24 h. The necrosis scores were significantly higher after PPVAL + ISS than after PVL or PPVL + ISS at all time points (*p* < 0.05). The necrosis scores of the PPVL + ISS group were lower than those of the other two groups at all time points (*p* < 0.05). *p < 0.05 compared with PPVL + ISS or PVL. ^#^*p* < 0.05 compared with PVL.

Necrosis scores from HE staining of the left median lobes at all time points were significantly higher after PPVAL + ISS than after PVL and PPVL + ISS (*p* < 0.05). The necrosis scores of the PPVL + ISS group differ significantly compared to the other two groups at all time points tested (*p* < 0.05) **(**Fig. 7**)**.

### Regenerating liver lobes showed up-regulation of cytokines mRNA expression

**Figure 8:**
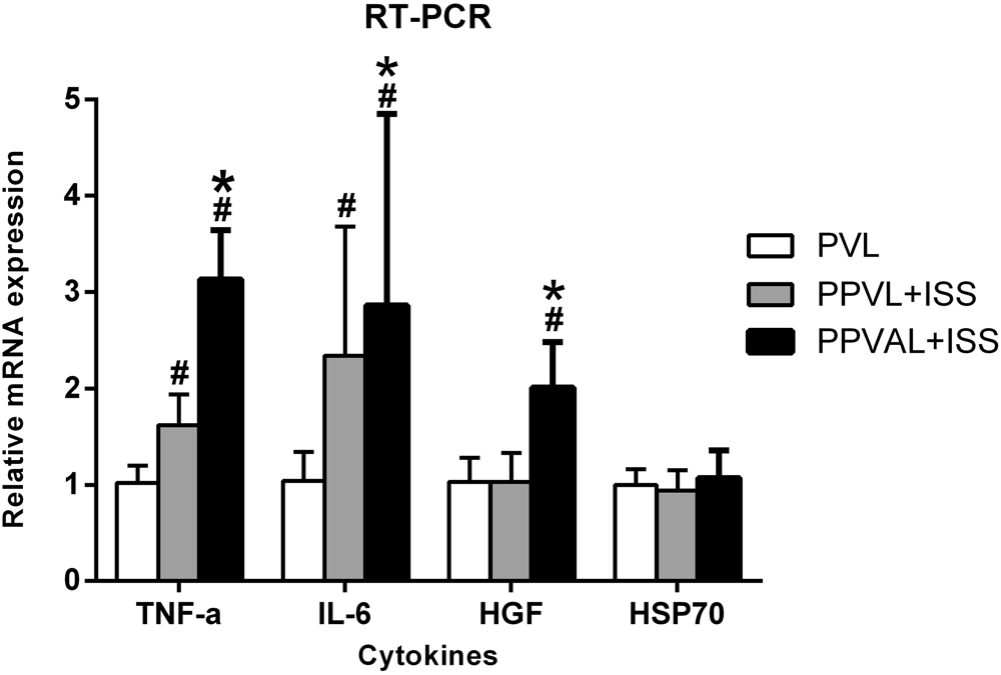
The mRNA expression of cytokines in the regenerating liver lobes at 24 h after surgery. The mRNA expression levels of TNF-α, IL-6 and HGF showed significant differences among these three groups. However, there was no significant difference in the mRNA levels of HSP70 among these three groups (*p* > 0.05). ^#^*p* < 0.05 compared with PVL. *p < 0.05 compared with PPVL + ISS.

A large number of cytokines are believed to have a close relationship with liver regeneration. The gene expression of these cytokines was up-regulated in the regenerating liver lobes. The mRNA levels of TNF-α, IL-6, HGF and HSP70 in the regenerating lobes at 24 h after surgery were measured. The expression of TNF-α and IL-6 were vastly up-regulated in the PPVAL + ISS group and PPVL + ISS group compared to those in the PVL group (*p* < 0.05) with the PPVAL + ISS group presenting significantly higher values than the PPVL + ISS group (*p* < 0.05). In the PPVAL + ISS group, mRNA levels of HGF were significantly higher compared with the PPVL + ISS group and PVL group (*p* < 0.05). The levels of HSP70 mRNA did not differ among all the three groups (*p* > 0.05) **(**Fig. 8**)**.

## Discussion

ALPPS was largely superior to PVE/PVL because of the latter’s flaws, which include a long waiting period before the second operation and inadequate liver regeneration in some patients who cannot meet the needs of surgery^16^. However, the first operation of ALPPS as well as PVE/PVL may also cause focal hepatic necrosis or tumor progression in the occluded lobe. In this study, we planned to evaluate the liver regeneration of the non-occluded lobe and hepatic necrosis of the occluded lobe following selective partial portal vein ligation or simultaneous concomitant hepatic artery ligation combined with *in situ* splitting in order to find ways to overcome the abovementioned limitations of PVE/PVL.

We observed an obvious decrease of the blood flow through the left portal branch after partial portal vein ligation as measured by ultrasound flowmeter. However, the blood flow through the left portal branch showed no significant change after the concomitant hepatic artery was ligated. The microcirculation of the right median lobes increased after PPVL. No obvious change occurred before and after concomitant HAL. The microcirculation of the left median lobes decreased after PPVL and showed additional obvious decreases after concomitant HAL. These results indicated that concomitant HAL had no significant effect on the blood flow through the left portal branch but had a significant influence on the microcirculatory blood perfusion of the left median lobes, which was a direct cause of the aggravated necrosis of left median lobes after concomitant HAL.

We focused on the HRR in the right median lobe following different surgical interventions in this study. Although the PPVL + ISS group showed a slightly lower HRR at 72 h and 7 d than that of the PVL group, the HRR between the PPVL + ISS and PVL groups had no significant differences at all time points. However, the HRR of the PPVAL + ISS group was obviously increased comparing with that of the other two groups at all time points except 24 h after surgery. This observation indicated that concomitant HAL could obviously promote remnant liver regeneration, which was also confirmed by the results of the Ki-67 immunostaining assay. The number of hepatocytes in the regenerating liver lobe positive for Ki-67 per visual field at 48 h and 72 h in the PPVAL + ISS group was greater than those in the other two groups. The number of hepatocytes positive for Ki-67 at 72 h in the PPVL + ISS group was lower compared to the PVL group, which was consistent with the relatively lower HRR in the PPVL + ISS group than in the PVL group.

Aggravated injury of the occluded lobe was also obvious after concomitant HAL. The necrotic area of left median lobes observed in HE staining in the PPVAL + ISS group were larger than that in the other two groups due to decreased microcirculation of the left median lobe following concomitant HAL. However, there was no large hepatic necrosis or liver abscess observed. This result indicated that inducing remnant liver regeneration using selective partial portal vein ligation and simultaneous concomitant hepatic artery ligation combined with *in situ* splitting is feasible and safe. In contrast with PPVAL + ISS, PPVL + ISS could ameliorate injury of the occluded lobe through increased portal and hepatic artery blood flow. The results of the HE staining showed there was occasional small spots of hepatic necrosis in the left median lobes from the PPVL + ISS group. The hepatic enzymes (ALT and AST) levels, an indication of hepatocellular injury, were obviously increased in the PPVAL + ISS group and were relatively low in the PPVL + ISS in the early stage after surgery, which was consistent with the results of the HE staining. The increased serum TBIL levels also demonstrated severe hepatic injury in the PPVAL + ISS group. Serum ALB levels were decreased in each group because of inflammatory and stress responses caused by surgery. The serum ALB concentrations of the PPVAL + ISS group were obviously increased at 72 h; we speculated that this was the result of rapid compensatory liver regeneration of the contralateral lobe.

We detected the mRNA levels in the regenerating lobes in order to clarify the relationship between cytokines and the different HRRs in three groups. TNF-α, IL-6 and HGF expression with PPVAL + ISS were all obviously up-regulated comparing with those in the other two groups, which could explain the accelerated liver regeneration after PPVAL + ISS. The expression of HSP70 showed no significant difference among the three groups, which suggested that the stress response did not play a significant role in the process of accelerated liver regeneration.

The blood flow through hepatic artery compensatorily increased after segmental portal vein embolization or ligation, which is known as the hepatic arterial buffer response (HABR). Gregoire *et al*.^23^ confirmed that HABR occurred when 20% stenosis of the portal vein was performed in an experiment focused on the different degrees of PPVL in pigs. Yokoyama *et al*.^24^ found hepatic arterial flow becomes the primary supply of the sinusoids in rats following restriction of the portal vein flow via partial portal vein ligation, and the blood perfusion of sinusoids decreased significantly after the concomitant hepatic artery was ligated. This observation was consistent with the results of our experiment, which showed that hepatic necrosis was aggravated after concomitant HAL. Increased hepatic arterial blood flow was also considered an important contributor to rapid tumor growth in the occluded lobe.

Some strategies such as transcatheter arterial chemoembolization (TACE)^25^ were adopted to restrict tumor progression in the occluded lobe after PVE/PVL, whereas chemotherapy^26^ was performed before PVE/PVL. However, implementation of either therapy needed an extended waiting period before the second operation and whether chemotherapy exerted toxic or adverse effects on liver regeneration was not clear yet. In this study, we have confirmed that PPVAL + ISS promoted obvious remnant liver regeneration while completely blocking hepatic arterial blood flow, which would inhibit tumor growth due to the loss of an arterial blood supply.

## Conclusions

In conclusion, combining ISS with selective PPVL or simultaneous HAL were both beneficial to liver regeneration. The HRR of PPVL + ISS was equivalent to that of PVL, and the injury of the occluded lobes from PPVL + ISS was less pronounced when compared to the lobes from PVL. Both the HRR and hepatic necrotic area with PPVAL + ISS was significantly increased comparing with PVL. However, no large areas of macroscopic necrosis appeared in the occluded lobes. Therefore, this combined procedure is safe and feasible. The results of the RT-PCR indicate that PPVAL + ISS promotes the release of inflammatory cytokines that are related to superior hepatic regeneration. We consider that PPVL + ISS has some clinical value for patients who have a mildly poor liver function or whose hepatic artery has become stenotic or occluded due to the invasion of lesions. Additionally, PPVAL + ISS may cause atrophy or necrosis of the tumor in the occluded lobe due to complete loss of the hepatic artery blood supply, which may provide a new approach for treating individuals with rapid tumor progression in the occluded lobe after PVE/PVL. Of course, the primary shortcoming of this study is the animals used in the experiment have normal liver tissue. However, most patients with hepatocellular carcinoma (HCC) also have comorbid cirrhosis. Therefore, further experiments in animals with cirrhosis and HCC are needed to clarify how to perform PPVAL (e.g., the degree of blood flow reduction through the left portal branch after the concomitant hepatic artery is ligated) to not only obtain sufficient hepatic regeneration but also ensure safety in animals. In addition, PPVAL + ISS could both effectively inhibit the growth of the tumor in the occluded lobe while simultaneously inducing hypertrophy of the contralateral lobe.
